# Testing and Assessing Method for the Resistance of Wood-Plastic Composites to the Action of Destroying Fungi

**DOI:** 10.3390/ma14030697

**Published:** 2021-02-02

**Authors:** Anna Wiejak, Barbara Francke

**Affiliations:** Building Research Institute, Filtrowa 1, 00-611 Warsaw, Poland; a.wiejak@itb.pl

**Keywords:** wood-plastic composites, methods of testing resistance to fungi, methods of assessment

## Abstract

Durability tests against fungi action for wood-plastic composites are carried out in accordance with European standard ENV 12038, but the authors of the manuscript try to prove that the assessment of the results done according to these methods is imprecise and suffers from a significant error. Fungi exposure is always accompanied by high humidity, so the result of tests made by such method is always burdened with the influence of moisture, which can lead to a wrong assessment of the negative effects of action fungus itself. The manuscript has shown a modification of such a method that separates the destructive effect of fungi from moisture accompanying the test’s destructive effect. The functional properties selected to prove the proposed modification are changes in the mass and bending strength after subsequent environmental exposure. It was found that intensive action of moisture measured in the culture chamber of about (70 ± 5)%, i.e., for 16 weeks, at (22 ± 2) °C, which was the fungi culture, which was accompanying period, led to changes in the mass of the wood-plastic composites, amounting to 50% of the final result of the fungi resistance test, and changes in the bending strength amounting to 30–46% of the final test result. As a result of the research, the correction for assessing the durability of wood-polymer composites to biological corrosion has been proposed. The laboratory tests were compared with the products’ test results following three years of exposure to the natural environment.

## 1. Introduction

In recent years greater attention has been paid to the aspects of sustainable development in construction. It is assumed that buildings are designed, constructed, and dismantled to enable sustainable use of natural resources, ensures the building structures’ long life, and allows environmentally friendly raw and secondary materials. On the one hand, there is a drive to develop materials that are more susceptible to biodegradation processes [1], but on the other hand, works are carried out on improving the durability of building materials. Although accelerated biodegradation of different types of materials improves the effectiveness of waste management, the drive to protect building materials against the impact of microorganisms results from two factors. First and foremost, the corrosion which occurs due to biodegradation may pose a hazard to the life and health of the users of these materials because of the toxic influence of fungi [2,3]. The other aspect means the need to ensure the required durability of the materials, which has a tremendous impact on their appearance. The issue applies to wood, too. Natural fibers have low densities and are biodegradable, and they are highly available and cheap, making them more attractive than traditional synthetic fibers. Still, there are some disadvantages of using these fibers, i.e., low thermal stability, susceptibility to moisture absorption, and biological degradation [4]. Wood-plastic composites [5], called WPC in short, used for terrace surfaces and wall cladding, result from the efforts to develop a product whose performance combines the advantages of wood and plastics [1]. The polymer material in WPCs is the matrix, whereas particles of plant origin are the filler. Typical polymer matrices include polyethylene, polypropylene, polyvinylchloride, and high-density polyethylene (HDPE), whereby polypropylene (PP) is most prevalent in Poland. The fillers include but are not limited to various lignocellulose-based particles, usually wood, in the form of flour, fibers, or fine chips. Other fibers of plant origin such as rice hulls [6] are also used. The process of fiber modification results in the end product being less capable of water absorption. It is crucial from the point of view of resistance to biological factors that develop on a damp substrate [7,8]. The decay can be caused by fungi, which develop favorable conditions for their growth [9]. Optimum environmental conditions mean humidity measured in the culture chamber of ca. 70%, adequate temperature, and pH ranging from 5.6 to 6.5 [10]. Wood-plastic composites are more resistant to biodeterioration than untreated wood; however, several laboratory and field studies have shown that the wood in these materials remains susceptible to decay for example Silva et al. [11], Morris and Cooper [12], Mankowski and Morrell [13], Pendleton et al. [14], Verhey et al. [15], and Schirp et al. [16]. Mentioned reports indicate that decay does occur, but destruction rates are slower than found with untreated wood of the same species. While moisture levels eventually reach the point at which biological attack is possible, the wetting rate is slow and most of the moisture is confined to a zone within 5 mm of the WPC surface [11,17]. Wang and Morrell [18] stated that the rate of moisture sorption and ultimately decay rate depends on the wood’s particle size and geometry, wood/polymer ratio, and the presence of other compounds that may repel water. Ibach et al. [17], in contrast to the information mentioned above, have done clinical magnetic resonance imaging and found a significant amount of water distributed randomly all across the board cross-sections of the decayed WPC, including the core of the samples. They explained that this phenomenon was caused by both the transport of moisture by fungal mycelia to a particular area of activity and by the generation of water as decay fungi metabolize wood, releasing bound water.

The development of specific fungi species depends on the substrate’s character and properties where they develop (the content of minerals, salination) [19]. The polymer material’s susceptibility to biodegradation processes results, e.g., from the polymer’s chemical structure and molecular weight, its physical and chemical characteristics, and the kind and intensity of the microorganisms’ impact [20,21]. Some literature findings indicate that thermoplastic material fully closes the composite’s wooden component, protecting it from moisture and decomposition caused by fungi, because synthetic polymers that typically demonstrate a lack or negligible susceptibility to biodegradation are the matrix of polymer composites [22]. It results, e.g., from the chemical structure and hydrophobic character of the material surface. Many papers suggest that natural fiber coating with a polymer matrix is incomplete [23,24]. It turns out that the fibers reach moisture content levels that enable fungi attack [25]. Schirp et al. [16] stated that if the wood filler’s moisture content can be kept out or at least below 20%, WPC decay may be prevented. This could be achieved by complete encapsulation of wood particles by the plastic matrix, hydrophobation of the WPC surface, or by chemical the wood substrate’s modification. They also stated that voids between wood and plastic represent entry points and proliferation pathways for microbes; thus, these should be eliminated or reduced. Morris and Cooper [12] were the first to prove the presence of touchwood and discoloration caused by fungi on WPC terrace decking profiles. In the conditions of use, terrace decking profiles made of wood-plastic composites are susceptible to simultaneous influence of biological corrosion, variable positive and negative temperatures in the presence of water and moisture, UV radiation, and additional mechanical functional loads [21,22]. Previous studies assumed that deterioration of the measured property by more than 50% disqualified the material for further use [26].

Of course, when evaluating the tests’ results, it is necessary to consider how they were performed. Biological corrosion tests are mainly performed using two methods. In North America, the soil-block test for wood [27,28,29] has been adopted for fungal durability tests of WPC in which weight loss serves as an indicator of decay. In Europe, the agar-block test, according to ENV 12038 [30], is commonly used in fungal decay testing. While American Standards [27,28] aims at determining the natural decay resistance of woods, ENV 12038 [29] is intended to determine the efficiency threshold of wood preservatives against wood-decay fungi. Schirp and Wolcott [30] compared the North American and European methods for WPC fungal durability testing. The agar-block test was modified such that no support rods were employed to accelerate moisture uptake by WPC specimens that had not been pre-treated, only steam-sterilized in an autoclave. It was determined that modified agar-and soil-block tests are equally suited for determining weight loss in WPC, but that agar-block tests can be completed in a shorter period. Basidiomycetes fungi are recommended in the method according to ENV 12038 [29]. The group Basidiomycetes fungi, mentioned above, belong among others white-rot decay *Coriolus versicolor* and brown rot decay fungi, *Coniophora puteana*. Lignin and cellulose fibers are the fungi’s food. *Coriolus versicolor* fungus attack lignin primarily, whereas *Coniophora puteana* fungus decomposes cellulose [31]. The mold growth is observed on composites; the mold uses contaminants on the surface as its food and reduces the material appearance rather than damage the material [32].

The aims of this manuscript are twofold: first to prove that the assessment of tests results obtained in tests done by European method used in fungal decay testing does not allow separate the destructive effect of fungi from the destructive effect of the moisture accompanying the test, second: propose a new method of assessing the test results. The research subject of the research was the decking WPC profiles used only on the ground’s surface, without contact with the soil; therefore soil-block method was not included in the tests. A fungus from the Basidiomycetes group called *Coniophora puteana* was the trigger of biological corrosion in the study. The presented tests were carried out in two stages. In the first stage, the changes in the reference material characteristics under the influence of the fungus mentioned above were determined in laboratory conditions. On the second stage, the assessment was extended to cover aging tests carried out in natural conditions for three years, i.e., including climate loads. The natural tests were carried out under climate load conditions corresponding to mid- European transitional climate Since the literature reports [4,5,20,23,33,34,35] suggested that the negative impact of destroying fungi might cause changes in the mechanical characteristics of the tested materials and a change in their weight, the change in the characteristics after the impact of aging factors was taken as a biodegradation measure; the research methodology intended to assess the characteristics was verified. The obtained results showed that intensive action of moisture of about (70 ± 5)%, i.e., for 16 weeks, at (22 ± 2) °C, which was accompanying the fungi culture period, led to changes in the mass of the wood-plastic composites, amounting to 50% of the final result of the fungi resistance test, and changes in the bending strength amounting to 30% ÷ 46% of the final test result.

## 2. Materials and Methods

### 2.1. Materials

Wood-plastic composites are primarily used in Poland for the production of decking profiles intended for balconies and terraces. That is why decking profiles were used for the tests. Since the most common solutions employed for the production of wood-plastic composites include polyethylene (PE), polypropylene (PP), polyvinyl chloride (PVC), and high-density polyethylene (HDPE), materials with such matrixes were used for the tests. Products with similar content of wood material were used in all tested cases.

The following materials were used in all tests:

➢ Sample I—decking profiles made of a composite containing wood flour (45%), polyvinyl chloride (PVC) (45%), pigments (5%), stabilizers, absorbents, fillers (5%),
➢ Sample II—decking profiles made of a composite containing wood flour (60%), HDPE plastic (30%), stabilizers and pigments (10%),
➢ Sample III—decking profiles made of composite with low content of wood flour (49%) and polypropylene (PP),
➢ Sample IV—decking profiles made of a composite containing wood flour (49%) and polyvinyl chloride (PVC) (51%).

Additionally, water absorption values of the samples I-IV are shown in Table 1. These values were determined after 14 and 28 days of immersing the samples in distilled water, at temperature (22 ± 2) °C. Mentioned characteristics are supplemented with samples’ water absorption values after removing fungi, i.e., after completion of the environmental exposures.

### 2.2. Environmental Exposure

**Figure 1:**
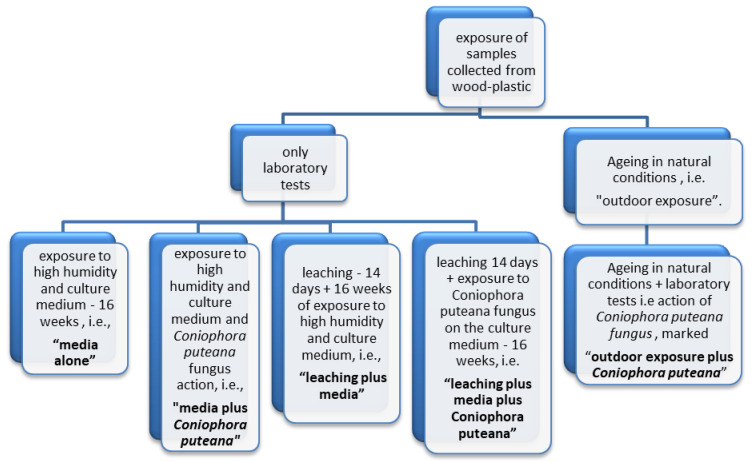
Schematic presentation of the samples of wood-plastic composite exposure patterns.

To determine the influence of destroying *Basidiomycetes* fungi, i.e., *Coniophora puteana*, on the durability of wood-plastic composites, compared to other functional impacts, the samples were exposed to various aging factors. *Coniophora puteana* fungus was selected because it is typically used in tests on wood protected with preservatives and it causes relatively large losses of the wood mass. That is why it was considered useful for the assessment of wood-plastic composites. The test samples were exposed according to the diagram shown in Figure 1.

**Figure 2:**
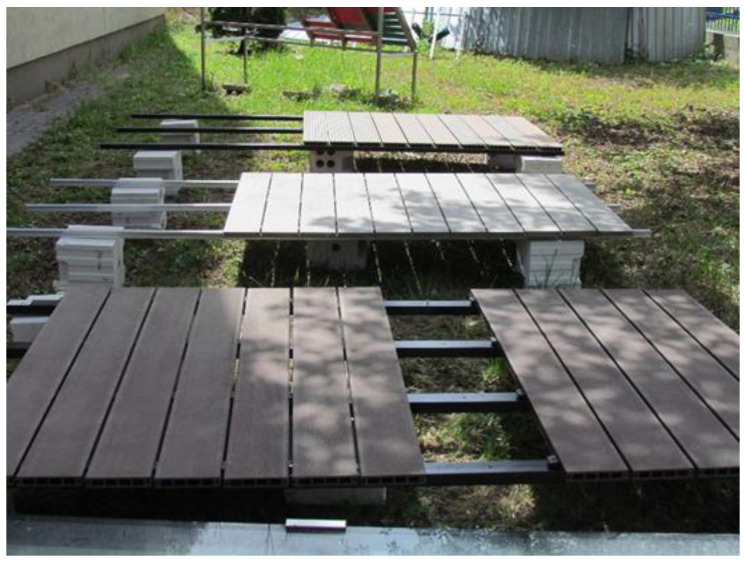
The view of wood-plastic decking profiles during exposure in natural conditions.

The exposures mentioned above involved:

- exposure to high humidity and culture medium—16 weeks (further marked as “media alone”). The samples are exposed in pairs in a Kolle flask on a culture medium, i.e., a substance composed of 40 g of malt extract, 35 g of agar, and water to 1000 mL at the test conditions at (22 ± 2) °C and (70 ± 5)% humidity, for 16 weeks. The medium (nutrient) is the food and source of moisture for the fungi during the test and simultaneously affects the test samples.
- leaching—14 days + 16 weeks of exposure to high humidity and culture medium (further marked as “leaching plus media”). The samples were immersed in water at (20 ± 2) °C for 14 days, according to the description given in EN 84 [36]. The samples were soaked in water, five volumes of water for one volume of sample, for two weeks. The water was replaced nine times during the cycle. After the end of the cycle, the samples were placed in flasks on maltose agar medium and incubated in the culture chamber at (22 ± 2) °C and (70 ± 5)% for 16 weeks. The leaching aging test according to EN 84 [36] is obligatorily used in tests of impregnated wood for exterior applications;
- exposure to high humidity and culture medium, and *Coniophora puteana* fungus action (further marked as “media plus *Coniophora puteana*”)—16 weeks. The samples were placed in Kolle flasks in two, on glass plates on the culture medium completely covered with fungus *Coniophora puteana.* The flasks with the samples were placed in the culture chamber and incubated for 16 weeks at (22 ± 2) °C and (70 ± 5)% humidity’
- leaching 14 days + exposure to *Coniophora puteana* fungus on the culture medium—16 weeks (further marked as “leaching plus media plus *Coniophora puteana*”). The samples were leaching in water for 14 days and then were placed in Kolle flasks in two, on glass plates on the culture medium completely covered with fungus *Coniophora puteana*. The flasks with the samples were placed in the culture chamber and incubated for 16 weeks at (22 ± 2) °C and (70 ± 5)% humidity;
- aging in natural conditions (further marked as “outdoor exposure”). Three mocks made of decking profiles screwed to composite ground beams were prepared in the test station in natural conditions, on the supports made of hollow bricks. After three years of exposure, the test samples were cut out from the profiles. The arrangement of the samples during the test in natural conditions is shown in Figure 2.
- aging in natural conditions and action of *Coniophora puteana fungus* (further marked as “outdoor exposure plus *Coniophora puteana*”). After exposure in natural conditions for three years, test samples were cut out from the profiles and placed in test vessels on the medium covered with *Coniophora puteana* fungus and exposed in a culture chamber for 16 weeks, as described above.

All types of composite decking profiles selected for the tests were exposed according to the first three patterns. Aging tests in natural conditions were carried out only for samples marked as sample III and sample IV.

Ten samples (10 mm × 80 mm, 5 mm thick) cut out from the face profiles’ surface any type of material (I-IV) were exposed according to each of the exposures listed above.

Simultaneously with the test samples, control samples were also used to determine the fungus activity. Ten virulens control test specimens (50 mm × 25 mm × 15 mm) with Scots pine wood were placed in Kolle flasks in two, on glass plates on the culture medium completely covered with fungus *Coniophora puteana* and were incubated like research samples. The loss in mass of the samples was obtained 38% ÷ 45% with the required ≥20%.

### 2.3. Methods of Tests

#### 2.3.1. Testing the Change in the Mass

Before exposing the samples to the aging process, their mass was determined. The samples cut out from the decking profiles were seasoned in a climatic chamber at (20 ± 2) °C and (65 ± 5)% humidity until obtaining a constant mass. Eight samples of each type of WPC material were dried in an oven at (103 ± 2) °C to constant mass and the initial dry matter (m_o_) of the test samples was calculated. The samples were exposed according to the patterns mentioned above. Following the exposure to fungus, the surface of the samples was cleaned of the mycelium. Once the aging processes were completed, the samples were dried at (103 ± 2) °C, and the end dry mass (m) of the samples was determined. The loss of the samples’ mass was the measure of the material decay caused by each exposure and was calculated based on the following Formula (1):

U = (m_o_ − m) × 100/m_o_ (1)

For all tested samples the assessment was carried out after the following exposures:

- exposure to high humidity and culture medium—16 weeks,
- exposure to high humidity and culture medium and *Coniophora puteana* fungus action—16 weeks,
- leaching—14 days + 16 weeks of exposure to high humidity and culture medium,
- leaching 14 days + exposure to *Coniophora puteana* fungus on the culture medium—16 weeks.

Additional tests were carried out for samples III and IV after aging in natural conditions and for three years, followed by exposure for 16 weeks in high humidity conditions on the medium overgrown by fungus.

#### 2.3.2. Bending Strength Tests

The bending strength tests of the samples following their exposure to aging factors were carried out according to EN ISO 178 [37], using eight samples of each material and for each test option. The tests were carried out at (23 ± 2) °C once different samples’ exposure patterns were completed. The samples’ surfaces were cleaned of any surface mycelium and dried to constant mass at (20 ± 2) °C and (65 ± 5)% humidity before the tests. The bending strength of the samples not subjected to aging (σ) was additionally identified to determine the change in the bending strength after exposure to aging processes (σ_i_), according to the Formula (2):

Z_σ_ = [(σ_i_ − σ)/σ] × 100 (2)

The tests were carried out on INSTRON strength tester after:

- exposure to high humidity and culture medium—16 weeks,
- leaching—14 days + 16 weeks of exposure to high humidity and culture medium,
- leaching 14 days + exposure to Coniophora puteana fungus on the culture medium—16 weeks.

Additional tests were carried out for samples III and IV after:

- aging in natural conditions,
- aging in natural conditions for three years, followed by 16 weeks of exposure to fungus on the culture medium.

## 3. Results

Table 2 summarizes the percent change in the mass after exposure to, sequentially: leaching, exposure to fungus, leaching and exposure to fungus, and three years of exposure in natural conditions at later exposure to the fungus.

**Figure 3:**
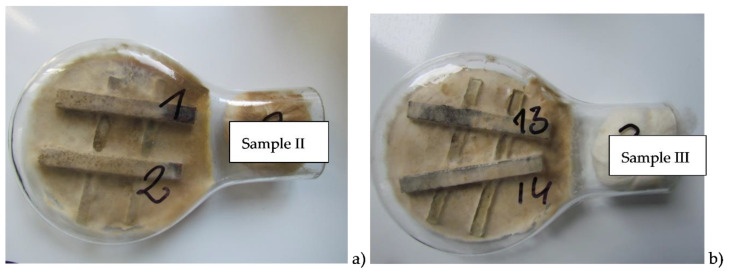
Samples made of the wood-plastic composite after 16 weeks of exposure on Coniophora puteana fungus (**a**) sample II, (**b**) sample III.

Figure 3 shows samples of wood-plastic composites after 16 weeks of exposure to *Coniophora puteana* fungus.

Table 3 summarizes the bending strength test results for the exposed samples.

## 4. Discussion

**Figure 4:**
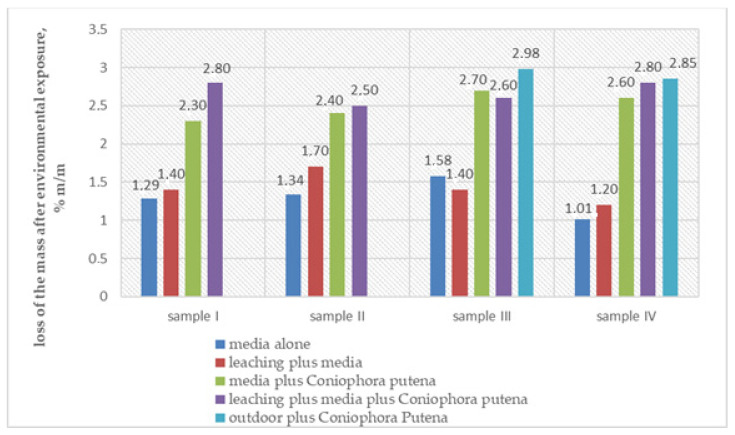
Influence of environmental exposure on the loss of mass in wood-plastic composites.

Figure 4 shows the percent changes in the samples’ mass in reference to their original mass, resulting from different exposure patterns. The obtained results suggest that exposure in high humidity conditions of ca. (70 ± 5)% at (22 ± 2) °C in the presence of culture medium causes a significant change in the mass (weight) of wood-plastic composite samples already during testing the material susceptibility to biological corrosion. The change in the mass of wood-plastic composites following long-term exposure to increased humidity amounts to 1.31% on average and increases slightly, within the measurement error range, if the samples are soaked in water for 14 days before the exposure. In the latter case, the mean loss of the mass is 1.35%.

Further comparison of the effects of exposure to different factors leads to a conclusion that the mean loss of the mass caused by exposure to fungus, following the exposure on the culture medium, amounts to 2.5%, whereby 50% of the value, i.e., 1.31%, is the negative influence of the test conditions, namely high humidity in the presence of the medium. The presented calculation suggests that the loss of the mass caused by the fungus’ actual impact amounts to 1.19%. The same is true for another factor—exposure to fungus after subjecting the samples to a long-term water impact. In this case, the mean values of the change in the mass after combined exposure to all the factors mentioned above amounts to 2.68%, which means that the fungus influence on the destructive process is 1.33% for the value of the loss in the wood-plastic composite mass.

The decay mechanism of WPC is often described in the literature [38] as the formation of a network of larger voids mainly through the connection of microvoids that are inherently present in the material. The mentioned process occurs as a result of an action of moisture alongside fungal activity or without the presence of fungi, only in moist conditions combined with elevated temperature. It was proved [39] that conditioning not only supplies the moisture needed for fungal growth but also effectively creates larger voids, assumably formed by the interconnection of smaller voids, which accelerates the decay process.

The exposure to natural use factor has shown to have no significant impact on the *Coniophora puteana* fungus growth. This has been confirmed by the observations made by Sun et al. [39], showing that the period of 3 years of storage of WPC in natural conditions is insufficient for fungal growth on the surface because of the low water absorption. They found out that in the field, an extensive time was required to initiate fungal colonization that results in wood weight loss, which may explain the deficiencies in some laboratory evaluations of WPC for fungal decay resistance [39]. Considering the above, samples after being exposed to natural conditions were exposed to Coniophora under laboratory conditions for 16 weeks. It seems that the influence of the fungus on the samples previously exposed to natural atmospheric conditions for 3 years on the increase in the loss of wood-plastic composite mass is minor. The comparison of the same samples subjected to natural aging and then exposed to the fungus on the sample medium with the samples exposed only to the fungus on the culture medium in laboratory conditions clearly shows that the increase in the loss of the mass following environmental exposure amounts to 0.27%, which means it is within the measurement error range.

**Figure 5:**
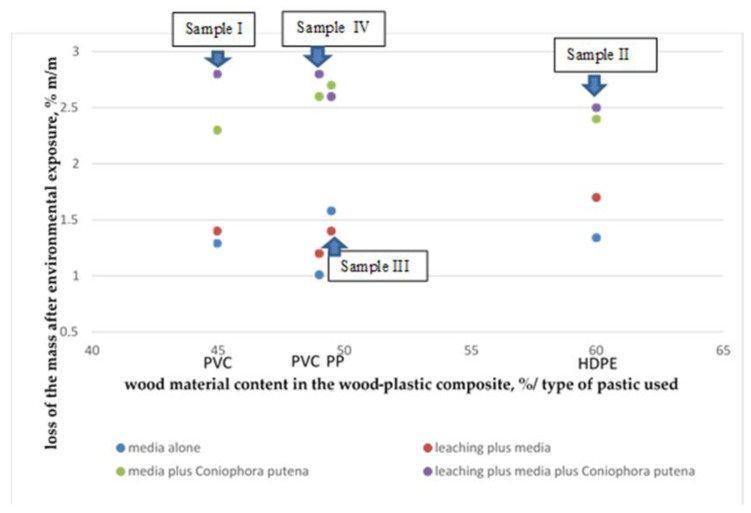
Comparison of the losses of mass after environmental exposure compared with the wood material content in the composite and polymer type in the matrix.

The obtained values of the loss of mass after exposure to aging factors were compared with the content of wood flour in the composite. Figure 5 shows the graphic representation of the values.

The summary reveals that the increase in the wood flour quantity has no significant influence on the composite susceptibility to the loss of mass due to biological corrosion, and the wood flour is the ingredient most sensitive to such exposure effects. Unfortunately, this conclusion does not confirm previous literature reports [13], but the obtained result may be affected by stabilizers, the composition of which is not known to the authors of the manuscript. The most significant losses of mass following the exposure to *Coniophora puteana* fungus were discovered for decking profiles with the polyvinyl chloride matrix, even though in both cases the content of the wood flour was the lowest among all tested products. The obtained results indirectly confirm the previous literature findings [19] that the thermoplastic material in a polypropylene and high-density polyethylene matrix closes the composite’s wooden component and thus protects it from moisture and decay caused by fungi.

**Figure 6:**
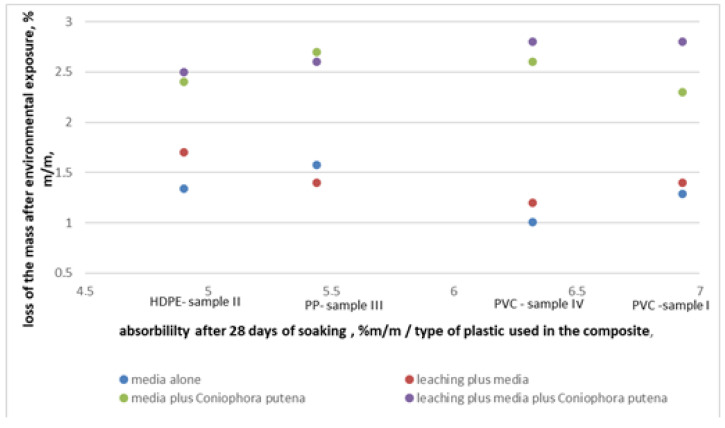
Comparison of the losses of mass after environmental exposure compared with the composite absorbability and polymer type in the matrix.

The results obtained for a polyvinyl chloride matrix (samples I and IV) are not so unequivocal. Sample I and sample IV are characterized by the highest absorbability values and they are accompanied by the loss of mass after exposure to environmental factors (Figure 6).

The observations mentioned above from laboratory studies confirm the observation related to the product’s behavior during natural conditions. Continuous dampness, i.e., using the product in shaded areas, with limited air circulation, at a simultaneous water accumulation possibility, often contributed to the surfaces being overgrown with house fungi. Not only does it deteriorate the appearance but may also cause a loss of the product’s performance.

The presented tests confirmed that the destructive action of *Coniophora puteana* fungus in the environments mentioned above, observed as a loss of mass, has a minor impact on the product life and should not contribute to the damage discovered during regular inspections of the building substance. Comparing the results with the limit values of the acceptable loss of mass for impregnated wood amounting to 3% reveals that the composites fall within the range, even if the influence of higher humidity exposure during the test on the final results is not eliminated.

**Figure 7:**
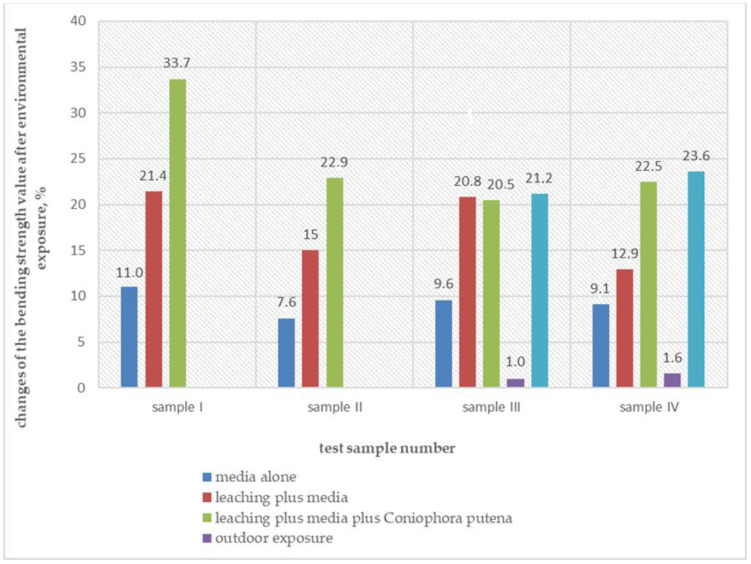
Reduction in the bending strength of wood-plastic composites after environmental exposure.

In light of the above, the gradual loss of the tested material’s strength can be even more worrying. The test composites’ bending strength was tested on the following stage of the study, after their environmental exposure (Figure 7). It is evident that the negative impact of the environment with higher humidity of ca. (70 ± 5)% in the presence of the culture medium has an equally strong influence on reducing the bending strength of wood-plastic composites and on their loss of mass. In all studied cases, the test products’ bending strength decreased from 7.6% up to 11% in the environment with (70 ± 5)% humidity, which amounts to 30–46% of the total test result. This action is intensified when the medium exposure in high humidity conditions is preceded by 14 days of immersion in water. It reduced the bending strength from 12.9% to 21.4%. In both presented cases the medium is the food for the fungi and maintains a specific moisture level. The obtained values of the bending strength reduction after the exposure mentioned above should not pose any significant problems related to the maintenance of structures made of the tested products within the evaluated range of functional loads. These findings are consistent with the results of studies and other scientists. Ibach et al. [38] also showed that the major factors degrading the mechanical properties of the tested composite under detected field exposure conditions were elevated temperature and moisture exposure, whereas UV radiation from the sun had a low impact, if any, on the flexural properties of a WPC board exposed for almost 10 years.

Thus, it seems justified to eliminate the influence of the exposure condition on the test result final assessment.

Taking the above into consideration, the authors proposed to apply Equation (3) for the evaluation of the wood-plastic composite susceptibility to the action of destroying fungi, defined by the change in the mass and bending strength after environmental exposure; it was also suggested to neglect the negative influence of the initial, 16 weeks’ exposure to the high humidity of (70 ± 5)%, at (22 ± 2) °C, in the presence of the medium.

Z_mg_ = (Z_x_ − Z_p_) (3)

where:

Z_mg_—loss of mass/bending strength reduction as a result of exposure to house fungi, %
Z_x_—loss of mass/bending strength reduction after combined exposure of all studied aging factors, %
Z_p_—loss of mass/bending strength reduction after exposure to high humidity environmental conditions—(70 ± 5)%, at 22 ± 2 °C, in the presence of the medium, %.

The proposal is also justified by changes in the wood-plastic composite bending strength after three years of exposure to natural environmental conditions. After the exposure mentioned above, the changes in the bending strength value did not exceed 2%. Once the samples were exposed to the fungus, and consequently the exposure on the culture medium, the obtained bending strength values were similar to those for the samples subjected to washing out in laboratory conditions for 14 days, followed by 16 weeks of exposure to the fungus. The same is true for the assessment of the change in the wood-plastic composite mass after environmental exposure. These observations conclude that for the assessment of wood-plastic composite resistance to *Coniophora puteana* fungus similar research outcomes can be obtained by substituting a long-term exposure cycle in the natural condition with a much quicker test carried out in the laboratory conditions.

## 5. Conclusions

The paper presents tests’ results on four randomly selected commercial wood-polymer composites exposed to environmental impacts related to *Coniophora puteana* fungi’s action on the reference products’ durability. Based on the obtained results, the following conclusions can be drawn:

- The proposed assessment method of wood-polymer composites resistance to destroying fungi introduces bending strength test as a supplement to the weight change assessment of samples exposed to environmental impacts both in the laboratory and natural conditions. The assessment of the bending strength decrease renders more accurate results than the method involving loss of mass because the coefficient of variation is lower for such series of results,
- The manuscript presents a modification of assessment of the resistance of wood-polymer composites to destroying fungi which allow one to separate the destructive effect of fungi from the destructive effect of the moisture accompanying the test. It was proved the negative impact of high humidity for 16 weeks constitutes a significant percentage of the test result. In the case of the mass change assessment, it exceeds 50%, whereas for the bending strength change assessment it ranges from 30% to 46%.
- The method of testing wood-plastic composites’ resistance to destroying fungi in laboratory conditions, presented in the paper, renders the results similar to those after three years of use in a natural environment and then infecting them with destroying fungi.

The performed tests confirm the high resistance of wood-plastic composites to destroying fungi.

## Figures and Tables

**Table 1:** Water absorption of samples I–IV.

Test Sample	Water Absorption, % m/m		
	After Immersion in Water for:		After Completion of Environmental Exposures (after Removal of the Fungi)
	14 Days	28 Days	
I	5.78	6.93	16.98
II	3.47	4.90	15.40
III	5.44	4.14	16.01
IV	5.12	6.32	16.50

**Table 2:** Effect of combination of exposure over agar in Kollar flask, 14 days of leaching, and 16 weeks of exposure to *Coniophora puteana* on mass loss of selected wood plastic composites.

Test Sample	Average Mass Loss after Exposure to % m/m/Coefficient of Variation [%]				
	Media Alone	Leaching Plus Media	Media Plus *Coniophora puteana*	Leaching Plus Media Plus *Coniophora puteana*	Outdoor Exposure Plus Media Plus *Coniophora puteana*
I	−1.29/(13.0)	−1.4/(6.4)	−2.3/(18.7)	−2.8/(17.9)	-
II	−1.34/(35.8)	−1.7/(28.2)	−2.4/(3.8)	−2.5/(19.6)	-
III	−1.58/(10.1)	−1.4/(25.7)	−2.7/(5.6)	−2.6/(5.4)	−2.98/(13.8)
IV	−1.01/(8.9)	−1.2/(2.5)	−2.6/(5.4)	−2.8/(5.4)	−2.85/(10.2)

**Table 3:** Effect of combination of exposure over agar in Kollar flask, 14 days of leaching, and 16 weeks of exposure to *Coniophora puteana* on mean bending strength values of selected wood plastic composites.

Test Sample	Bending Strength after Exposure: MPa/Coefficient of Variation [%]					
	Original	Media Alone	Leaching Plus Media	Leaching Plus Media Plus *Coniophora puteana*	Outdoor Exposure	Outdoor Exposure Plus Media Plus *Coniophora puteana*
I	36.5/(8.8)	32.5/(4.6)	28.7/(6.6)	24.2/(10.7)	-	-
II	35.4/(11.0)	32.7/(5.5)	30.1/(3.7)	27.3/(4.4)	-	-
III	29.3/(2.7)	26.5/(3.4)	23.2/(4.3)	23.3/(7.3)	29.0/(6.6)	23.1/(3.4)
IV	36.4/(4.1)	33.1/(6.3)	31.7/(2.2)	28.2/(7.1)	35.8/(5.9)	27.8/(4.7)

## Data Availability

https://biblioteka.itb.pl.

## References

[1] Prochoń M., Witczak M., Biernacka A. (2017). Wood as a Component of Polymer Biocomposites (In Polish: Drewno Jako Składnik Biokompozytów Polimerowych).

[2] Andersson M.A., Nikulin M., Koljalg U., Andersson M.C., Rainey F., Reijula K., Hintikka E.L., Salkinoja-Salonen M. (1997). Bacteria, molds, and toxins in water-damaged building materials. Appl. Environ. Microbiol..

[3] Fisher M.C., Henk D.A., Briggs C.J., Brownstein J.S., Madoff L.C., McCraw S.L., Gurr S.J. (2012). Emerging fungal threats to animal, plant and ecosystem health. Nature.

[4] Tascioglu C., Goodell B., Lopez-Anido R., Peterson M., Halteman W., Jellison J. (2003). Minitoring fungal degradation of E-glass/phenolic fiber reinforced polymer (FRP) composites used in wood reinforcement. Int. Biodeter. Biodegrad..

[5] Eder A., Carus M. (2013). Global trends in wood-plastic composites (WPC). Bioplast. Mag..

[6] Penczek S., Pretula J., Lewiński P. (2013). Polymers from renewable raw materials, biodegradable polymers (in Polish: Polimery z odnawialnych surowców, polimery biodegradowalne). Polimery.

[7] Orhan Y., Buyukgungor H. (2000). Enhancement of biodegradability of disposable polyethylene in controlled biological soil. Int. Biodeterior. Biodegrad..

[8] Leja K., Lewandowicz G. (2010). Polymer biodegradation and biodegradable polymers—A review. Pol. J. Environ. Stud..

[9] Gautam R., Bassi A.S., Yanful E.K. (2007). A review of biodegradation of synthetic plastic and foams. Appl. Biochem. Biotechnol..

[10] Wołejko E., Matejczyk M. (2011). Civil and Environmental Engineering Reports. Bud. I Inżynieria Środowiska.

[11] Silva A., Gartner B.L., Morrell J.J. (2007). Towards the development of accelerated Methods for Assessing the Durability of wood Plastic Composites. J. Test. Eval..

[12] Morris P.I., Cooper P. (1998). Recycled plastic/wood composite lumber attacked by fungi. For. Prod. J..

[13] Mankowski M., Morrell J.J. (2000). Patterns of fungal attack in wood plastic composites following exposure in a soil block test. Wood Fiber Sci..

[14] Pendleton D.E., Hoffard T.A., Addock T., Woodward B., Wolcott M.P. (2002). Durability of an extruded HDPE/wood composite. For. Prod. J..

[15] Verhey S., Laks P., Richter D. (2001). Laboratory decay resistance of woodfiber/ thermoplastic composites. For. Prod. J..

[16] Schirp A., Ibach R.E., Pendelton D.E., Wolcott M.P. (2008). Biological Degradation of Wood-Plastic Composites (WPC) and Strategies for Improving the Resistance of WPC against Biological Decay.

[17] Ibach R., Sun G., Gnatowski M., Glaeser J., Leung M., Haight J. (2015). Exterior decay of wood–plastic composite boards: Characterization and magnetic resonance imaging. For. Prod. J..

[18] Wang W., Morrell J.J. (2004). Water sorption acharacteristics of two wood-plastic composites. For. Prod. J..

[19] Nuhoglu Y., Oguz E., Uslu H., Ozbek A., Ipekoglu B., Ocak I., Hasenekoglu I. (2006). The accelerating effects of the microorganisms on biodeterioration of stone monuments under air pollution and continental-cold climatic conditions in Erzurum. Turk. Sci. Total Environ..

[20] Coffta G., Borysiak S., Doczekalska B., Garbarczyk J. (2006). Resistance of polypropylene-wood composites to fungi (in Polish: Odporność kompozytów polipropylen-drewno na rozkład powodowany przez grzyby). Polimery.

[21] Falk R., Lundin T., Felton C. The Effects of Weathering on Wood-Thermoplastic Composites Intended for Outdoor Applications. Proceedings of the 2nd Annual Conference Durability and Disaster Mitigation in Wood-Frame Housing.

[22] Faruk O., Błędzki A.K., Fink H.P., Sain M. (2012). Biocomposites reinforced with natural fibers: 2000–2010. Prog. Polym. Sci..

[23] Kamdem D.P., Jiang H., Cui W., Freed J., Matuana L.M. (2004). Properties of wood plastic composites made of recycled DHPE and wood Flour from CCA- treated wood removed from service. Composites Part A.

[24] Yildiz U.C., Yildiz S., Gezer E.D. (2005). Mechanical properties and decay resistance of wood polymer composites prepared from fast growing species in Turkey. Bioresour. Technol..

[25] Clemons C.M., Ibach R.E. (2004). Effects of processing method and moisture history on laboratory fungal resistance of wood-HDPE composites. Prod. J..

[26] Sobków D., Barton J., Czaja K., Sudoł M., Mazoń B. (2014). Reasearch on the resistance of materials to the effects of the natural environment (in Polish: Badania odporności materiałów na działanie czynników środowiska naturalnego). Chemik.

[27] ASTM D-2017 (2005). Standard Test Method for Accelerated Laboratory Test of Natural Decay Resistance of Wood.

[28] ASTM D-1413 (2007). Standard Test Method for Wood Preservatives by Laboratory Soil-Block Cultures.

[29] ENV 12038 (2002). Durability of Wood and Wood-Based Products—Wood-Based Panels—Method of Test for Determining the Resistance against Wood-Destroying Basidiomycetes.

[30] Schirp A., Wolcott M.P. (2005). Influence of fungal decay and moisture absorption on mechanical properties of extruded wood-plastic composites. Wood Fiber Sci..

[31] Seefeldt H., Braun U. (2012). Burning behavior of wood-plastic composite decking boards in end-use conditions; the effects of geometry, material composition and moisture. J. Fire Sci..

[32] Naumann A., Seefeldt H., Stephan I., Braun U., Noll M. (2012). Material resistance of flame retarded wood-plastic composites against fire and fungal decay. Polym. Degrad. Stab..

[33] Chmielnicki B., Jurczyk S. (2013). WPC composites as an alternative to wood products (in Polish: Kompozyty WPC jako alternatywa dla wytworów z drewna). Przetwórstwo Tworzyw.

[34] Ashori A., Behzad H.M., Tarmian A. (2013). Effects of chemical preservative treatments on durability of wood flour/HDPE composites. Composites.

[35] Friedrich D., Luible A. (2016). Investigations on ageing of wood-plastic composites for outdoor applications: A meta-analysis using empiric data derived from diverse weathering trials. Constr. Build. Mater..

[36] EN 84 (1997). Wood Preservatives. Accererated Ageing of Treted Wood Prior to Biological Testing. Leaching Procedurę.

[37] EN ISO 178 (2019). Plastics—Determination of Flexural Properties.

[38] Sun G., Ibach R.E., Faillace M., Gnatowski M., Glaeser J.A., Haight J. (2017). Laboratory and exterior decay of wood–plastic composite boards: Voids analysis and computed tomography. Wood Mater. Sci. Eng..

[39] Ibach R., Gnatowski M., Sun G., Glaeser J., Leung M., Haight J. (2018). Laboratory and environmental decay of wood–plastic composite boards: Flexural properties. Wood Mater. Sci. Eng..

